# Research on the application of deep learning-driven urban change detection in sustainable development of hilly-area cities in western China

**DOI:** 10.1038/s41598-026-53767-y

**Published:** 2026-05-24

**Authors:** Haiying Wang, Mingzhong Wu

**Affiliations:** 1 School of Electronic Information Engineering, China West Normal University, Nanchong, 637000 China; 2 School of Mathematical Sciences, China West Normal University, Nanchong, 637000 China

**Keywords:** Deep learning, Urban change detection, Sustainable development, Environmental sciences, Environmental social sciences, Geography, Geography

## Abstract

Urban sustainable development is a critical pathway to overcoming resource constraints in the urbanization processes and achieving synergistic high-quality economic development and ecological security in hilly areas city of western China. This study investigates the application of deep learning-driven urban change detection technology in supporting sustainable urban development, taking Nanchong, Sichuan Province—a typical hilly areas city in western China as the case study area. Three core tasks were conducted. First, region-adapted urban element datasets were constructed, including a building and road semantic segmentation dataset (BR_Data_NC) and a change detection dataset (CD_Data_NC), both tailored to the landscape characteristics of hilly urban areas. These datasets provide targeted and reliable support for training and validating deep learning models suitable for medium-resolution remote sensing imagery in hilly regions. Second, deep learning models were applied to conduct semantic segmentation of buildings and roads based on BR_Data_NC, and further performed urban change detection using CD_Data_NC. The experiments were carried out entirely on the self-constructed datasets to ensure reasonable evaluation under consistent data characteristics. Comparative experiments demonstrated that deep learning-driven change detection effectively addresses challenges in complex hilly urban environments, such as fragmented landscapes, scattered buildings, and spectrally mixed features. Third, leveraging the change detection outcomes, this study analyzed urban expansion patterns in the research area, uncovering the evolutionary characteristics and potential trends in urban spatial morphology. The findings indicate that deep learning technology offers a robust tool for dynamic urban monitoring and informed decision-making in the context of sustainable urban development in hilly areas cities of western China. This approach exhibits clear practical value for optimizing urban spatial structure, improving land-use efficiency, and supporting coordinated urban development.

## Introduction

Sustainable development carries profound strategic significance for global urban development. The 2030 Agenda for Sustainable Development, adopted by 193 United Nations member states in 2015, established a comprehensive framework to promote urban cooperation centered on inclusiveness, safety, and resilience. However, amid the rapid advancement of human society, the tension between global urbanization and the achievement of the Sustainable Development Goals (SDGs) has intensified. According to the UN’s 2024 Sustainable Development Goals Report, only 17% of the SDG targets are on track, while nearly half are either stagnant or regressing. Urbanization is a key driver of this divergence^1^. Each year, global urban expansion consumes approximately 3 million hectares of natural habitats-equivalent to losing an ecosystem the size of New York City every hour^2^. Concurrently, urbanization has deepened social inequality: around one billion urban residents live without access to safe housing, and essential public services such as education and healthcare remain unevenly distributed. Poverty and food insecurity are especially acute in slum areas^3^. Furthermore, conventional urban planning models lack adaptability and fail to enable real-time monitoring of economic, environmental, and social dynamics, leading to delayed policy responses. Although the SDGs explicitly call for inclusive, safe, resilient, and sustainable cities, the trajectory of global urbanization continues to deviate significantly from these objectives, underscoring the urgent need for innovation in technological approaches and policy instruments^4^.

The future population growth in mountainous and hilly areas will mainly concentrate in small and medium-sized towns. Urban expansion is likely to have a significant impact on fragile ecosystems. However, in global land cover analysis, mountainous and hilly towns are often overlooked due to the low spatial resolution of satellite images and the difficulty in identifying fragmented settlements, resulting in significant deficiencies in the monitoring of urban spatial distribution and expansion dynamics in complex terrain areas. Chen et al. focused on the Hindu Kush-Himalaya region and verified the potential of deep learning in the detection of towns at the sub-pixel scale in mountainous areas, and explored the impact of temporal features on the detection results. They confirmed that deep learning models represented by U-NET are more suitable for extracting small towns and fragmented construction areas in complex terrain^5^. The terrain in the western part of China’s hills is complex, with fragmented surfaces, small and scattered feature patches, and scattered distribution. It faces similar problems to mountainous town monitoring. Zhao et al. proposed the MADNet change detection network for multi-scale features and dynamic sampling, by adjusting the receptive field adaptively, using multi-scale dilated convolution and enhanced attention mechanisms, effectively improving the extraction ability of the edges and texture features of fragmented targets, and achieving precise reconstruction of high-resolution feature maps, providing technical references for change detection in complex hills^6^.

In hilly areas, human activities and land use changes have a profound impact on the regional development pattern. Waqas et al. combined remote sensing change detection and multi-criteria decision analysis techniques to conduct an assessment of urban-rural land conflicts and a suitability analysis of cultivated land, and found that hilly areas generally face common problems such as rapid urban expansion, significant loss of cultivated land, fragmented land use layout, and sharp human-land conflicts^7^. Chakrabarty et al. took the Ayodhya hilly area in India as an example and conducted change detection based on multi-temporal remote sensing images, further confirming that both the construction of hydropower stations and urban development in hilly areas will trigger intense and large-scale land use conversions, directly altering the surface landscape pattern^8^. Wasim and Ravindra pointed out that such land use changes will also directly alter regional hydrological processes and surface erosion intensity, intensify ecological risks, and pose potential impacts on regional ecological security and sustainable development^9^. As a key spatial unit for urban development and ecological protection, hilly river basins generally face multiple contradictions between agricultural development, urban expansion, and ecological environment. Zhang et al. took the eastern hilly river basins in China as the object and compared and analyzed the response patterns of water environment under ecological engineering regulation, confirming that small watersheds with similar terrain conditions will show significantly different ecological evolution trends due to different human activity intensities and ecological intervention measures^10^. Barman et al. used the analytic hierarchy process to allocate weights for various environmental factors in the hilly towns of northeastern India, effectively identifying regional resource and environmental potential and spatial development patterns, providing methodological references for spatial control and planning decisions in hilly areas^11^.

Hilly area cities in western China face significant challenges in achieving coordinated development between ecological protection and urbanization due to the region’s complex geographical conditions and fragile ecological foundation. The transformation of urban and rural land use in the western hilly areas is intense, with processes such as abandoned farmland, urban expansion, and changes in construction land interweaving with each other. Wang and Song, using the hilly and gully area of the Loess Plateau as the research object, obtained long-term land use maps based on Landsat data and random forest classification. They constructed a change detection method through land use change trajectories to accurately extract abandoned farmland^12^. Duan et al. used a change detection method based on remote sensing time series data and machine learning classification to effectively capture the land use transformation process in the hilly area, providing a feasible path for monitoring urban expansion, identifying farmland loss, and analyzing changes in urban and rural land use^13^. Wang et al. conducted land cover change detection in the Xihai Ecological Demonstration Zone through supervised classification to assess the effectiveness of soil and water conservation measures, further demonstrating the significant application value of multi-temporal remote sensing classification and change detection in ecological governance and land use monitoring in the hilly areas^14^.

Traditional urban change detection mostly relies on manual visual interpretation or thresholding remote sensing image classification technology. When dealing with the processing of multi-phase, multi-source heterogeneous data in the complex geomorphic environment of hilly areas city in western China, there are problems such as low data accuracy, poor timeliness, and high cost. Manual interpretation relies on experience, has low repeatability and a long processing cycle, and is difficult to meet the requirements of dynamic detection^15^. Remote sensing image classification based on threshold is sensitive to category division and prone to accumulate classification errors, resulting in large detection errors^16^. Deep learning technology offers a new path to solve the above-mentioned predicament. Deep neural network (DNN) was proposed by Hinton et al.^17^. It is a machine learning process that acquires a multi-layer network structure based on sample data through training methods^18^. DNNS are classified into three types: feedforward deep networks, feedback deep networks, and bidirectional deep networks. The most widely used convolutional neural network (CNN)^19,20^ is a typical feedforward deep network, which is a trainable multi-layer network composed of multiple single-layer networks. Each single-layer network consists of three stages: convolution, nonlinear transformation, and downsampling^21^. Unlike traditional methods, CNN can automatically extract features and perform classification through sequence convolution and fully connected layers. Therefore, it can be regarded as a one-stage method that combines feature extraction and classification in a single model. CNN has become a core tool for semantic segmentation and change detection tasks. Semantic segmentation models based on CNN include AlexNet^22^, Visual Geometry Group (VGG)^23^, Fully Convolutional Networks (FCN)^24^, U-Net^25^, Inception^26^, ResidualNetwork (ResNet)^27^, and DeepLab v1 to DeepLab v3+^28–31^. Change detection models based on CNN include fully convolutional Siamese concatenation (FC-Siam-conc)^32^, dual-task constrained deep Siamese convolutional network (DTCDSCN)^33^, the combination of Siamese network and NestedUNet (SNUNet)^34^, bitemporal image transformer (BIT)^35^, attention-based multiscale transformer network (AMTNet)^36^, and transformer-based Siamese two-stream CD framework (ScratchFormer)^37^. However, most existing deep learning models are designed and validated primarily for high‑resolution remote sensing images, while applications and validations for medium‑resolution images remain insufficient. Such models are difficult to directly adapt to the spectral and spatial characteristics of medium‑resolution data, limiting their performance in regional‑scale urban change detection. Then, this study adopts representative and mature deep learning architectures to verify the feasibility and effectiveness of change detection using medium‑resolution remote sensing images.

Applying deep learning technology to the detection of urban changes in the hilly areas of western China can quickly and accurately extract information on changes such as land cover conversion and building alterations within the region. It is well-suited for the complex terrain, fragmented features, and scattered urban settlements in the western hilly areas. Combined with multi-temporal remote sensing images, the deep learning algorithm can efficiently identify changes in land cover types within the same geographical area, especially the subtle changes such as the construction and demolition of low-rise buildings and the expansion of scattered construction land that are unique to the western hilly cities. This provides timely and reliable data support for urban planning and spatial control in this region. Compared to traditional change detection methods, deep learning can effectively overcome the difficulties of fragmented terrain, spectral mixture of features, and shadow interference in the western hilly areas, improving the accuracy and efficiency of urban change identification in complex environments. This technology application not only provides key decision support for the promotion of new urbanization, the protection of historical cultural heritage, and the realization of the dual carbon goals in the western hilly areas, but also accurately captures the transformation patterns of urban and rural land use in the region, helping to solve the problems of urban development and ecological protection in this area, and injecting impetus into the sustainable development of the western hilly cities.

## Materials and methods

**Fig. 1:**
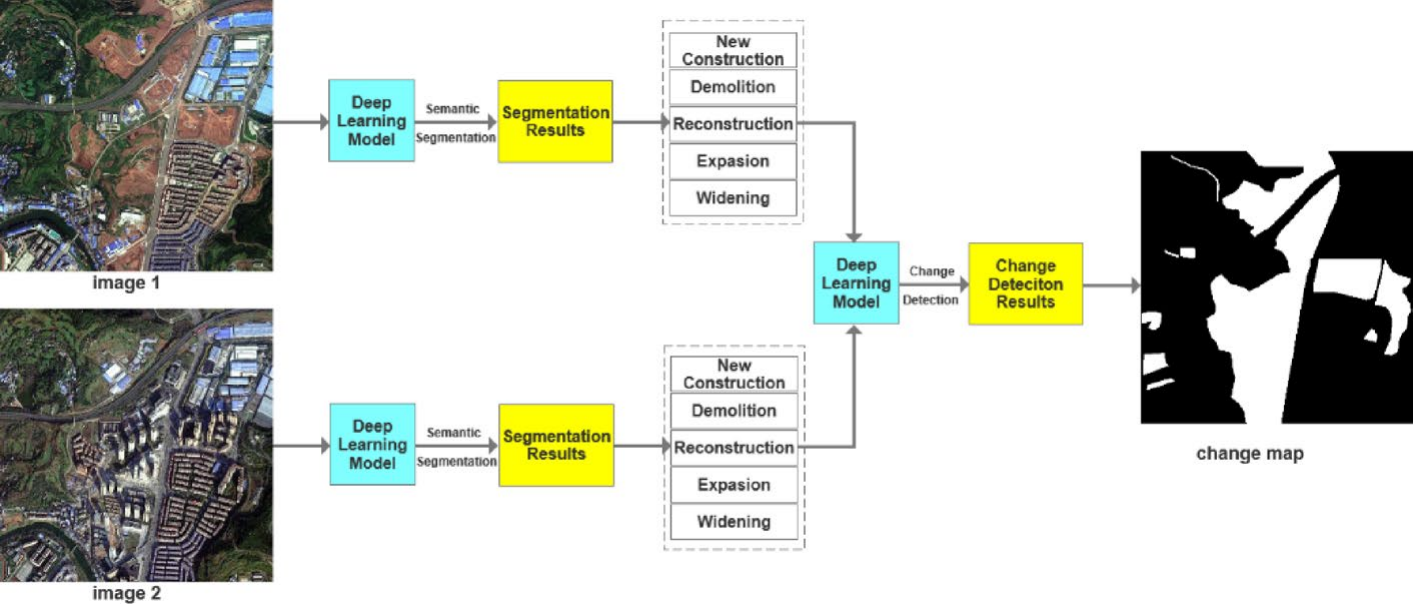
A framework for urban change detection based on semantic segmentation of buildings and roads.

Building and road extraction serves as a core technical approach for urban change detection. Deep learning models are employed to perform semantic segmentation on multi-temporal imagery of urban buildings and roads, enabling change detection through comparative analysis of segmentation results. Semantic segmentation is used for extracting static elements, while change detection is used for monitoring dynamic expansion, the overall framework is illustrated in Fig. 1. Building semantic segmentation allows for the precise extraction of urban building information. By comparing segmentation outputs across different time periods, specific building change types-such as new construction, demolition, and expansion-can be accurately identified, thereby providing essential data support for urban management, planning, and monitoring of urban dynamics. Road semantic segmentation facilitates the extraction of road networks. Comparing road segmentation results from different time phases enables the identification of road change types, including new construction, reconstruction, and widening, which supports road classification and the construction of road network maps. Finally, based on the accurate extraction of buildings and roads across multiple time points, the proposed method effectively identifies changes in urban spatial morphology and reconstructs transportation network topology, enabling comprehensive and dynamic urban change detection.

### Building semantic segmentation using deep learning

Architecture serves as the driving force behind urban expansion and constitutes a fundamental component of cities. It not only shapes urban development but also enhances urban prosperity. The construction of new buildings facilitates spatial expansion and helps alleviate congestion caused by population growth. Building renovation improves space utilization efficiency and supports sustainable urban growth. Architectural changes can transform a city’s appearance, enhancing its historical character and cultural distinctiveness, thereby attracting more tourists. Urban expansion relies on buildings for physical and functional support, while buildings themselves form the structural foundation of such expansion. Architecture provides essential residential, commercial, and public service facilities, creating economic opportunities and fostering broader urban development. Furthermore, it strengthens a city’s economic vitality, improves residents’ quality of life, and contributes to the enhancement of the urban environment.

In deep learning, building extraction is formulated as a semantic segmentation task, aimed at monitoring urban expansion and changes in building counts by extracting key information such as building contours, locations, and heights. Two main trends have emerged in building semantic segmentation. The first focuses on achieving highly precise building boundaries. Zhou et al. employed the Mask R-CNN model to detect buildings across multiple scales and achieved superior performance in capturing edge regions during segmentation^38^. Zhu et al. proposed the E-D-NET model, in which E-Net is responsible for capturing and preserving boundary details, while D-Net refines the output of E-Net to produce more accurate and detailed results. The second trend emphasizes the effective handling of buildings with diverse shapes and multi-scale characteristics^39^. Wu et al. utilized a multi-constraint FCN framework to optimize intermediate-layer parameters, thereby enhancing the model’s ability to represent multi-scale features^40^. Kang et al. introduced the EU-Net model, which incorporates dense spatial pyramid pooling to capture fine-grained details and improve the representation of building structures across varying scales^41^.

### Road semantic segmentation using deep learning

Roads serve as the foundation of urban expansion and the cornerstone of city, town, and village development. They facilitate transportation, resource distribution, and service delivery-functions that are essential for economic growth. Roads also enable access to employment opportunities, markets, education, healthcare, and broader social services. By improving urban traffic conditions, they contribute to enhanced mobility and stimulate economic development. The construction of new roads increases traffic capacity, improves traffic flow, accelerates travel speeds, shortens commuting times, and enhances travel efficiency, thereby boosting overall urban economic productivity. Additionally, new roads promote the development of adjacent residential and commercial zones, driving urban spatial expansion and creating new opportunities for urban growth. Furthermore, well-planned road infrastructure can improve the urban environment by reducing congestion-related pollution and enhancing residents’ quality of life.

Road extraction in deep learning is also formulated as a semantic segmentation task. By extracting key information such as road direction, width, and intersections, the road types can be identified and road network diagrams can be constructed. Fully Convolutional Network (FCN) is the most widely used architecture for road extraction. This method requires refined annotated urban element labeled samples to train the model effectively. To address the challenge of balancing weights in the image loss function, Zhang et al. proposed a comprehensive road extraction strategy based on spatial consistency, implemented within an FCN framework^42^. Chen et al. identified and integrated beneficial components from existing architectures to construct a lightweight network architecture for road extraction based on DenseNet^43^. Hou et al. introduced a remote sensing image road extraction method using a complementary U-Net approach. In their method, certain extracted regions were masked out using a fixed threshold, enabling the identification of complementary road segments in the erased areas. Multiple segmentation results were then fused to generate the final road extraction output^44^.

The RU-Net model proposed by Wang et al.^45^ was employed for semantic segmentation of urban buildings and roads. This model integrates an improved residual network into the U-Net architecture to reduce the number of network parameters and alleviate network degradation. Additionally, an atrous spatial pyramid pooling (ASPP) module is incorporated as a bridge unit between the encoder and decoder, enhancing the extraction and integration of multi-shape, multi-scale features, and contextual information. The model adopts focal loss (FL) instead of standard cross-entropy (CE) as the loss function to address the issue of class imbalance in urban image semantic segmentation.

### Change detection using deep learning

Deep learning primarily achieves change detection through deep neural networks (DNNs), enabling effective identification of spatial and temporal changes and delivering more accurate detection results. By leveraging the complex features extracted by DNNs, deep learning can automatically identify representative change patterns. Furthermore, recurrent neural networks (RNNs)^46^ can be employed to extract time-series change features, thereby enhancing change detection capabilities.

The deep learning model performs a comparative analysis of semantic segmentation results for buildings and roads across different time points to quantify changes in urban functional areas and construct dynamic spatiotemporal maps. In the context of remote sensing-based multi-temporal urban change detection, Annarita et al. analyzed ultra-high-resolution images using deep features extracted from pre-trained convolutional layers of AlexNet, which effectively captured contextual information^47^. Wang et al. reconstructed historical urban development and simulated future expansion patterns in the North China Plain-the world’s fastest-urbanizing region^48^. Iris et al. proposed a method based on a deep Siamese kernel point convolution network that enables direct change detection in 3D data. By integrating 2D image change detection with 3D point cloud analysis, their approach achieves simultaneous change detection and classification in a single step^49^. Wan et al. employed the Difference Guided Diffusion Model (DGDM) to enhance the alignment between image features and textual semantics, generating more accurate and detailed change detection outputs in building area monitoring^50^. Wu et al. integrated deep learning networks with Bayesian regularization to improve the accuracy and reliability of predictive models^51^. Yang et al. applied a backpropagation neural network to optimize commercial space utilization in the central urban area of Weinan City, China^52^.

The BiUnet-Dense model proposed by Wang et al.^53^ was employed for change detection of dual-temporal remote sensing images. This model constructs a dual-U-Net architecture to receive information inputs from two time phases. Additionally, to address the issue that the influence generated by layers that are far apart in the neural network’s backpropagation gradually decreases as the number of network layers increases, the model incorporates an encoder method based on long short-term memory networks. Finally, to solve the problems of increased network parameters due to dense connections, redundant backpropagation data, and significant increase in GPU consumption, the model employs a dense connection method by adding dropout layers after the convolutional layers.

## Dataset construction

### Basic information of the study area

Shunqing District, Nanchong City, Sichuan Province was selected as the study area to investigate the application of deep learning-driven urban change detection in supporting the sustainable development of cities in western China. Located in the northeastern part of Sichuan Province on the west bank of the Jialing River, Shunqing District lies in the central region of the Sichuan Basin (latitude 30.41°- 30.51°, longitude 106°- 107.07°). It serves as the political, economic, and cultural core of Nanchong City and functions as a key node in the high-quality development of the Chengdu-Chongqing Twin-City Economic Circle. The district covers a total area of 555 square kilometers and comprises 7 townships and 12 subdistricts, with a permanent population of 831,000, including 715,200 urban residents and 115,800 rural residents. The urbanization rate reaches 86.06%, reflecting a pronounced urban-rural dual structure.

The study area faces dual challenges of rapid urbanization and land resource constraints, which manifest in three key aspects. First, urban expansion monitoring: the built-up area has reached 95 square kilometers, and the development of the Linjiang New District continues to expand. Accurate identification of construction land boundaries is essential to prevent the non-agriculturalization of farmland. Second, renewal of the old urban area: the renovation of 965 old residential communities has been completed, benefiting 290,000 residents. Change detection is required to evaluate the effectiveness of these renovations and optimize subsequent project planning. Third, ecological protection pressure: initiatives such as the conservation of the Jialing River ecological corridor and the construction of a 13-kilometer riverside greenway necessitate continuous monitoring of environmental indicators including vegetation coverage and water quality.

### Dataset construction

**Fig. 2:**
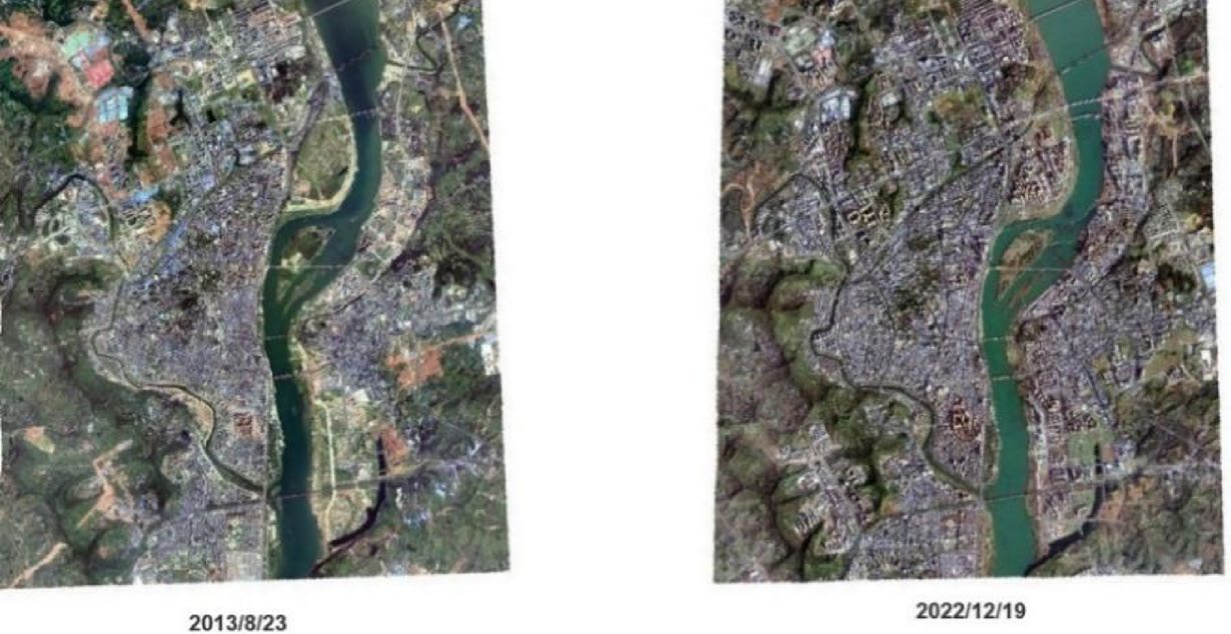
Two-phase remote sensing image maps of the study area.

Dataset creation involves data collection, cleaning and preprocessing, and annotation. Based on the objectives of urban feature semantic segmentation and change detection, the study area was narrowed to the main urban and street areas of Shunqing District, covering approximately 100 square kilometers and defined by the WGS84 coordinates: 106.15°E, 30.83°N; 106.07°E, 30.85°N; 106.02°E, 30.76°N; and 106.11°E, 30.73°N. The area exhibits diverse land use types, including buildings, roads, green spaces, water bodies, and bare soil. SPOT6/7 image data from August 23, 2013 and December 19, 2022 were used for the study. The original data include 1.5-meter panchromatic and 6-meter multispectral bands. In accordance with research requirements, the 6-meter multispectral data were selected for dataset construction, as shown in Fig. 2.

#### Building and road dataset (BR_Data_NC)

**Fig. 3:**
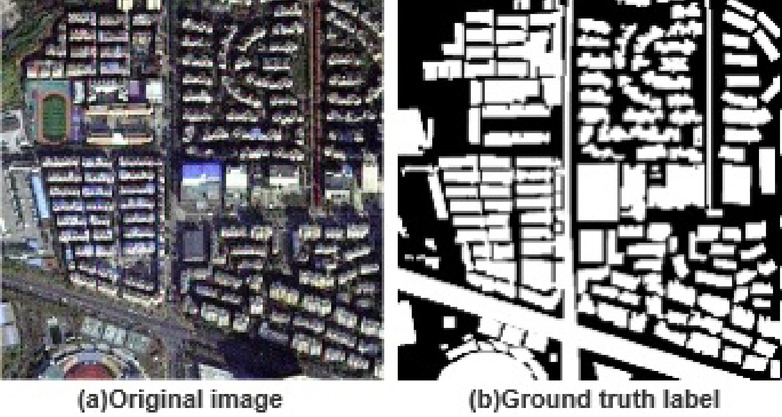
Example of the building and road dataset.

The building and road dataset is based on SPOT7 image data acquired on December 19, 2022, and includes diverse land cover features such as buildings, roads, squares, green spaces, water bodies, and bare soil. This dataset supports semantic segmentation of buildings and roads and can be applied to urban planning and construction, traffic optimization, and urban management efficiency improvement. The study area was partitioned into 168 non-overlapping image patches of size 512 × 512 pixels, with 100 images allocated for training, 35 for validation, and the remaining 33 for testing. Unlike the Massachusetts road dataset, which employs line-based annotations for roads, this dataset uses surface-level delineation for all roads, enabling a more accurate representation of their geometric shapes. An example of the BR_Data_NC dataset is shown in Fig. 3.

#### Change detection dataset (CD_Data_NC)

**Fig. 4:**
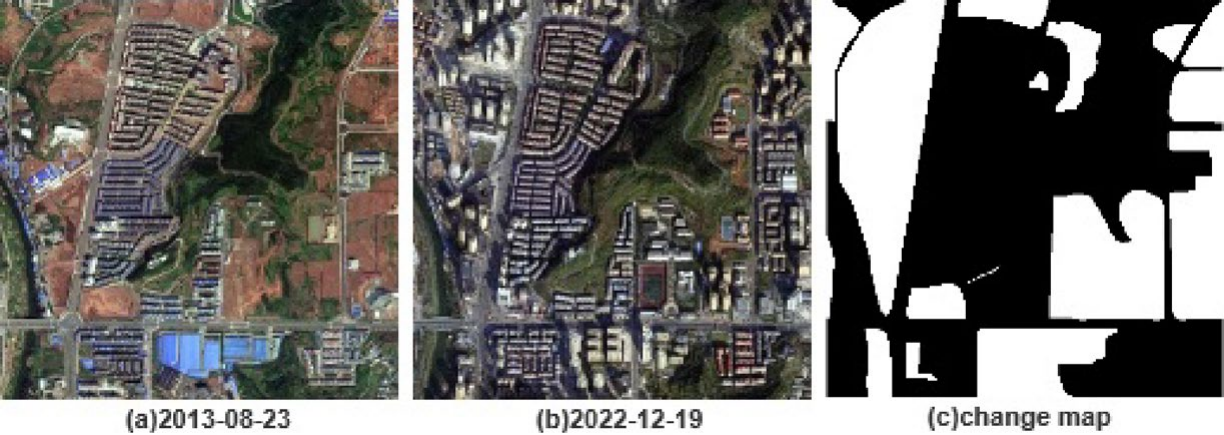
Example of the change detection dataset.

The change detection dataset is based on SPOT6 and SPOT7 images of the study area acquired on August 23, 2013 and December 19, 2022, respectively. Changes between the two images were manually labeled to construct the change detection dataset. To facilitate comparison, buildings and roads were comprehensively covered to ensure extensive spatial representation. The study area was divided into 48 non-overlapping image patches of size 1024 × 1024 pixels, with 35 images allocated for training, 7 for validation, and the remaining 6 for testing. An example of the CD_Data_NC dataset is shown in Fig. 4.

#### Data augmentation

Deep learning requires a large volume of sample data to train models and enable accurate predictions. Insufficient data can hinder the model’s ability to learn meaningful patterns and produce reliable outputs. Moreover, deep learning models are highly data-dependent, and their success relies on access to substantial training datasets; when the dataset is too small, effective model training becomes unfeasible. The original sample size was limited, mainly due to the cost of obtaining remote sensing images in the study area and the workload of sample annotation: The study area is located in the shallow hilly valley region of western China. The cost of purchasing 6-meter resolution remote sensing images is high, and it is objectively difficult to obtain long-term and comprehensive images. At the same time, the sample annotation for urban change detection tasks requires a combination of on-site verification and visual interpretation of images. Fine pixel-level annotations require a significant amount of manpower and time, resulting in an extremely large annotation workload, making it difficult to further increase the initial base sample size. To alleviate the problem of insufficient model training caused by the lack of basic samples, we performed data augmentation prior to training with the deep learning model to improve semantic segmentation performance.

Data augmentation is a widely adopted technique for enhancing dataset quality and diversity, typically achieved through geometric transformations, pixel-level modifications, and machine learning-based methods. In this study, geometric transformations were applied to augment both the BR_Data_NC and CD_Data_NC datasets. Through these transformations, the 168 original samples and corresponding labels in the BR_Data_NC dataset were expanded to 2016 new samples and labels, comprising 1200 training, 420 validation, and 396 test samples. Similarly, the 48 original samples and labels in the CD_Data_NC dataset were increased to 576 new samples and labels, including 420 training, 84 validation, and 72 test samples. The purpose of the data augmentation operation is to enhance the training stability and fitting effect of the model in the shallow hills and valleys of the western part of China, and to help the model better learn the characteristics of the landforms in the study area.

#### Comparison with classic public datasets

##### BR_Data_NC dataset

The BR_Data_NC building and road dataset was segmented using deep learning models such as U-Net, DeepLab v3+, and RU-Net. The performance of semantic segmentation was evaluated by comparing the metrics with those of the publicly available WHU building dataset, Inria aerial dataset, and Massachusetts road dataset. This research was implemented in the Keras API of the TensorFlow framework. During the training process, to increase the diversity of training samples, the same random slicing method was employed to resize the images to 256 × 256 size during each iteration. The model was trained and predicted on a server equipped with NVIDIA GeForce GTX 2080Ti and Intel(R) Xeon(R) CPU E5-2690 v4. The network hyperparameters were set as follows: 200 training iterations, batch size of 32, and learning rate of 0.001. The comparison results are presented in Table 1. In this context, Recall, Precision, F1, and IoU denote the recall rate, precision rate, harmonic mean of precision and recall, and intersection over union, respectively. Due to the higher complexity of the dataset data and more interference information, training on the BR_Data_NC dataset takes much longer compared to other datasets, being more than twice as long as the required time for other datasets. Nevertheless, the BR_Data_NC dataset achieved superior performance across all evaluation metrics (Recall, Precision, F1, and IoU) compared to the Massachusetts road dataset. Although there are slight performance differences compared to the WHU and Inria building datasets, BR_Data_NC still achieved high evaluation scores. It has strong adaptability and generalization ability for complex, irregular, and easily obstructed segmentation targets, thus providing a reliable data foundation for precise semantic segmentation in localized urban scenes.

**Table 1:** Evaluation metrics for semantic segmentation using deep learning models on different datasets.

**Dataset**	**Models**		
	**RU-Net**	**U-Net**	**DeepLab v3+**
	**Recall(%)** **/** **Precision(%)** **/** **F1(%)** **/** **IoU(%)** **/** **Time(s)**	**Recall(%)** **/** **Precision(%)** **/** **F1(%)** **/** **IoU(%)** **/** **Time(s)**	**Recall(%)** **/** **Precision(%)** **/** **F1(%)** **/** **IoU(%)** **/** **Time(s)**
BR_Data_NC	94.53/94.62/94.58/89.71/631	90.57/86.57/88.52/79.41/323	89.03/80.52/84.53/69.40/1819
WHU dataset	96.98/97.48/97.23/94.61/88	96.81/96.93/96.87/93.94/43	86.57/90.57/88.52/79.41/222
Inria dataset	93.73/96.14/94.92/90.33/120	94.72/94.86/94.78/90.11/64	92.52/96.31/94.38/89.35/365
Massachusetts road dataset	80.50/92.95/86.28/75.87/294	79.03/70.52/74.52/59.40/150	74.89/92.30/62.72/66.14/912

##### CD_Data_NC dataset

The FC-Siam-conc, SNUNet and BiUnet-Dense deep learning models were used to conduct change detection on the CD_Data_NC dataset. The performance of the change detection was evaluated by comparing various indicators with those of the publicly available change detection datasets OSCD and CD_Data_GZ. The deep learning models were implemented based on the nn.Module framework of Pytorch. During each iteration, each image was processed to a size of 32 × 32. The model’s hyperparameters were set as follows: the number of training iterations was 50, the batch size was 32, and the size was 96. The training was completed on a server equipped with NVIDIA GeForce GTX 2080Ti and Intel (R) Xeon (R) CPU E5-2690 v4. The evaluation metrics are presented in Table 2. Pre_c, Rec_c, Pre_nc, Rec_nc, F1, and Kappa denote the precision and recall for changed classes, precision and recall for unchanged classes, the harmonic mean of precision and recall, and the classification consistency between changed objects, respectively. The BiUnet-Dense achieved higher F1 values and Kappa coefficients in the OSCD dataset compared to the comparison models. It effectively suppressed complex background noise and accurately identified fine-grained changing features. Meanwhile, it maintained a balanced and stable precision and recall rate in the CD_Data_GZ dataset, without any obvious missed detections or false detections. In the CD_Data_NC dataset with complex features, the precision of the change area was ahead of other models, demonstrating a strong ability to extract multi-scale change features. Due to the rich scale of land features, large and small-scale change targets covered, and high data feature richness in the CD_Data_NC dataset, the training time was relatively long. Considering the comprehensive detection accuracy and robustness advantages, the CD_Data_NC dataset provides a solid and adaptable foundation for the dynamic monitoring of fine-grained urban elements in urban scenes.

**Table 2:** Evaluation metrics for change detection using deep learning models on different datasets.

**Dataset**	**Models**		
	**BiUnet-Dense**	**FC-Siam-conc**	**SNUNet**
	**Pre_c(%)** **/** **Rec_c(%)** **/** **Pre_nc(%)** **/** **Rec_nc(%)** **/** **F1(%)** **/** **Kappa(%)** **/** **Time(s)**	**Pre_c(%)** **/** **Rec_c(%)** **/** **Pre_nc(%)** **/** **Rec_nc(%)** **/** **F1(%)** **/** **Kappa(%)** **/** **Time(s)**	**Pre_c(%)** **/** **Rec_c(%)** **/** **Pre_nc(%)** **/** **Rec_nc(%)** **/** **F1(%)** **/** **Kappa(%)** **/** **Time(s)**
OSCD	43.22/57.13/97.93/96.09/49.21/46.18/416	35.29/25.59/96.18/97.56/29.67/26.62/395	34.21/27.64/93.28/95.02/32.95/29.15/213
CD_Data_GZ	58.25/56.03/94.36/94.83/57.14/51.74/140	69.06/45.25/93.24/97.38/54.68/50.18/132	67.46/50.58/90.98/93.85/48.10/44.22/75
CD_Data_NC	86.12/67.81/61.89/80.05/75.88/61.94/2255	63.84/66.28/65.03/49.09/73.78/52.18/2139	47.90/61.49/56.61/53.15/77.88/51.77/1156

## Experimental results and analysis

### Semantic segmentation of buildings and roads

**Fig. 5:**
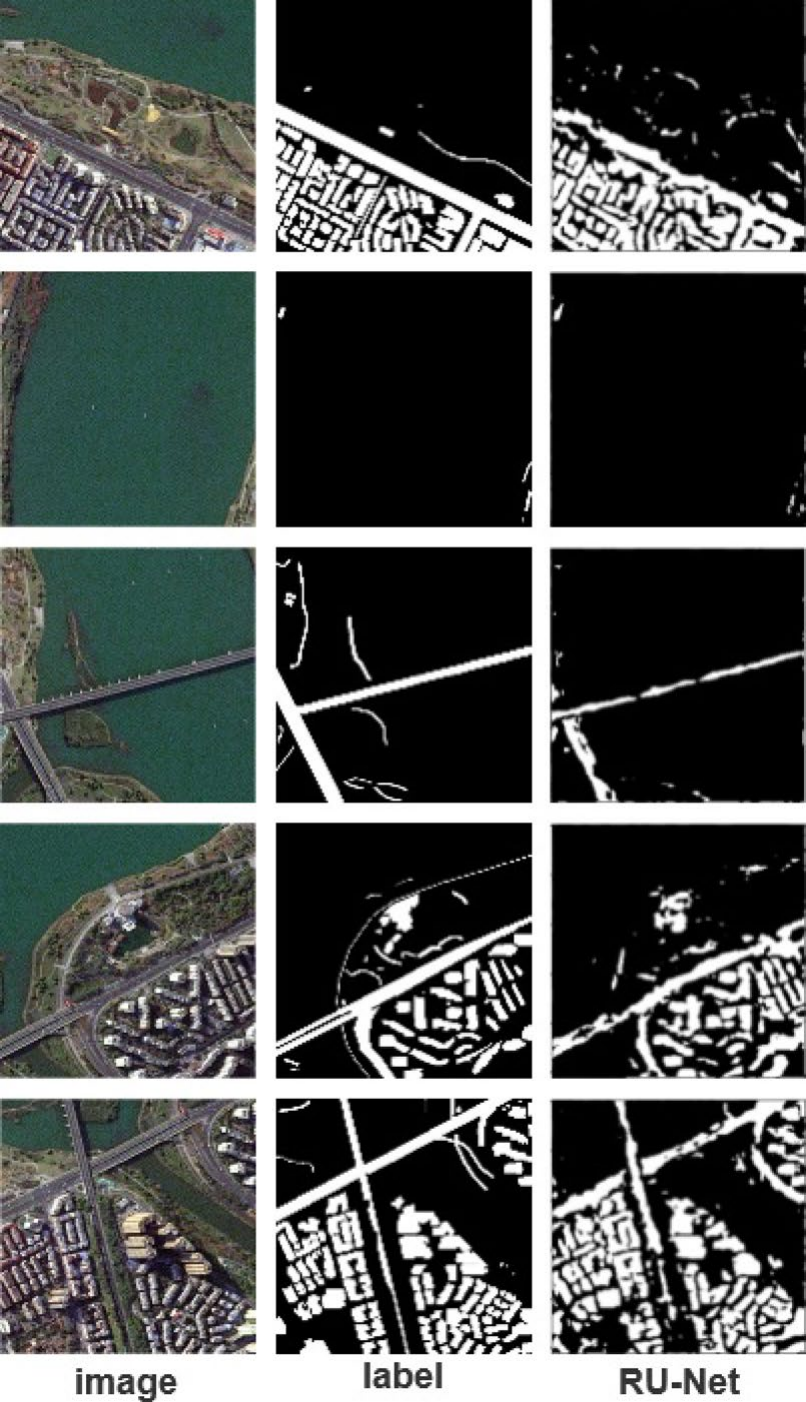
Semantic segmentation results of the BR_Data_NC dataset.

Figure 5 presents the semantic segmentation results of the RU-Net model on different scenarios in the BR_Data_NC dataset. The results demonstrate that the RU-Net model successfully segments the target features. However, due to the relatively low ground resolution of 6 m, the dataset poses inherent challenges for fine-grained delineation. Furthermore, segmentation accuracy is affected by factors such as illumination shadows, vegetation coverage, building height-induced occlusion, and labeling errors introduced during manual annotation. As a result, improvements are still needed in the delineation of building boundaries, road continuity, and overall spatial integrity.

The first row illustrates the identification of areas with closely spaced buildings. The RU-Net model successfully detects buildings and their approximate outlines; however, lighting conditions and small inter-building gaps significantly affect the accuracy of boundary delineation. The second row presents the identification of negative samples. Since the study area is in the core economic zone of Shunqing District, urban infrastructure such as buildings and roads dominates the landscape. Moreover, due to the nature of image segmentation, fully negative sample regions (i.e., areas devoid of target features) are absent from the dataset. Therefore, test data containing minimal building and road coverage were selected for evaluation. The results indicate that the RU-Net model performs well in identifying negative samples, with predictions generally consistent with the ground truth labels. The third row focuses on the detection of narrow or unpaved road branches. Due to the limited number of such road instances in the training data, the model’s overall capability to recognize fine-scale road segments remains insufficient. It can identify the general shape of wider roads but largely fails to detect narrower or unpaved branches. The fourth row shows results for simple scenes with low-rise buildings. Except for minor undetected road branches, both buildings and wider roads are accurately identified, with clear separation between structures. The fifth row demonstrates performance in complex scenes with high-rise buildings. The results show that the RU-Net model effectively handles mutual occlusion and shadowing caused by tall buildings, achieving high accuracy and producing well-defined boundaries.

It can be observed that the deep learning model achieves favorable performance in semantic segmentation of urban buildings on the BR_Data_NC dataset. The model can recognize complex scene structures, including building shapes, sizes, and spatial arrangements, and effectively handles occlusion relationships among high-rise buildings. However, its performance deteriorates in scenarios involving shadows and very narrow inter-building gaps. Regarding road semantic segmentation, the model exhibits slightly inferior results compared to building segmentation, primarily due to the limited number of road samples in the dataset and frequent partial occlusion of roads by vegetation in the labeled data.

### Urban change detection

**Fig. 6:**
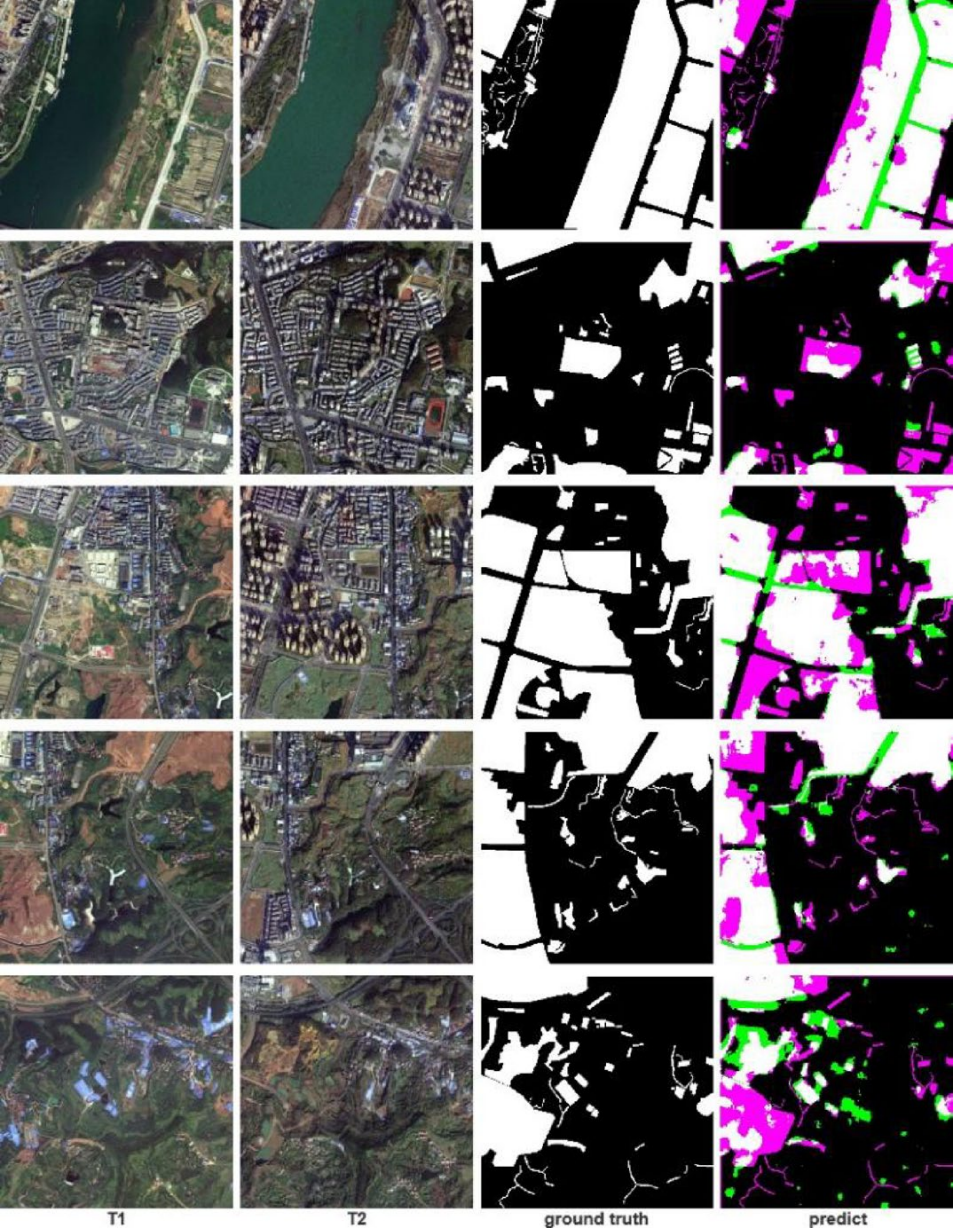
CD_Data_NC dataset change detection results.

Figure 6 shows the change detection results of the BiUnet-Dense model across different scenarios in the urban dataset CD_Data_NC. White denotes true positives (TP), black denotes true negatives (TN), green denotes false positives (FP), and magenta denotes false negatives (FN). TP indicates correctly identified changed areas, where positive samples are accurately predicted as positive by the model. TN refers to correctly identified unchanged areas, where negative samples are correctly classified as negative. FP represents incorrectly detected changes, corresponding to negative samples misclassified as positive. FN indicates missed changes, referring to positive samples erroneously predicted as negative.

The results show that the model successfully identifies many true positive (TP) cases, demonstrating high accuracy and well-defined boundaries. The predicted shapes across the five scenarios are largely consistent with the ground truth label data. However, the model misclassifies urban roads affected by lighting conditions or shadowed by tall buildings as false positives (FP). In addition to these incorrect road predictions, certain changes are missed and classified as false negatives (FN). The frequent occurrence of FN can be attributed to several factors: roads covered by vegetation in rural areas or green spaces, narrow roads, unpaved dirt roads, mutual shading between vegetation-covered or high-rise buildings, seasonal variations in surface vegetation coverage, and bare soil surfaces. Notably, a careful comparison of the dual-temporal images reveals that not all FN instances represent actual prediction errors. In the first row, the region labeled as changed in the reference data corresponds to an area partially constructed in the T1 image and completed by T2; thus, the model’s prediction more accurately reflects the ongoing construction process. In the second, third, and fourth rows, vegetation is largely restored, new buildings emerge, and bare soil decreases significantly. While the entire region is annotated as changed in the label set, the model’s output captures the change patterns with greater spatial precision. Nevertheless, some limitations remain in the change detection results. In the fifth row, isolated buildings with no actual change are incorrectly predicted as FP.

## Urban expansion patterns and analysis

### Urban expansion patterns

The newly added construction land in Shunqing District of Nanchong City generally exhibits a spatial distribution pattern characterized by extending along the valley, expanding towards the river, expanding in groups, and being guided by transportation. It is mainly located in the flat areas and gentle slopes of the Jialing River valley and the adjacent shallow hills. It extends along the transportation routes and the riverfront areas in an axial band-like manner, presenting a pattern of evolving from a single center to a multi-center group structure. The newly added land is mainly allocated in the flat areas of the Jialing River valley, the first and second terraces, and the gentle slopes of the shallow hills. The high hills and steep slopes generally have no large-scale new construction. This reflects the typical expansion pattern of a shallow valley city. In terms of spatial structure, it shows a multi-node concentration. The old urban area mainly focuses on urban renewal and the revitalization of existing resources. The newly added land is fragmented and scattered, and the newly built areas expand in contiguous and concentrated forms. Along the river and along the riverbank areas, the development intensity is high, with distinct strip-like features. The expansion along the transportation corridor is prominent, with the newly added land concentrated on both sides of the main arterial road networks, presenting a corridor-like and axial band-like expansion. Transportation hubs and main roads play a strong guiding role in the spatial distribution.

#### Expansion to flat areas

**Fig. 7:**
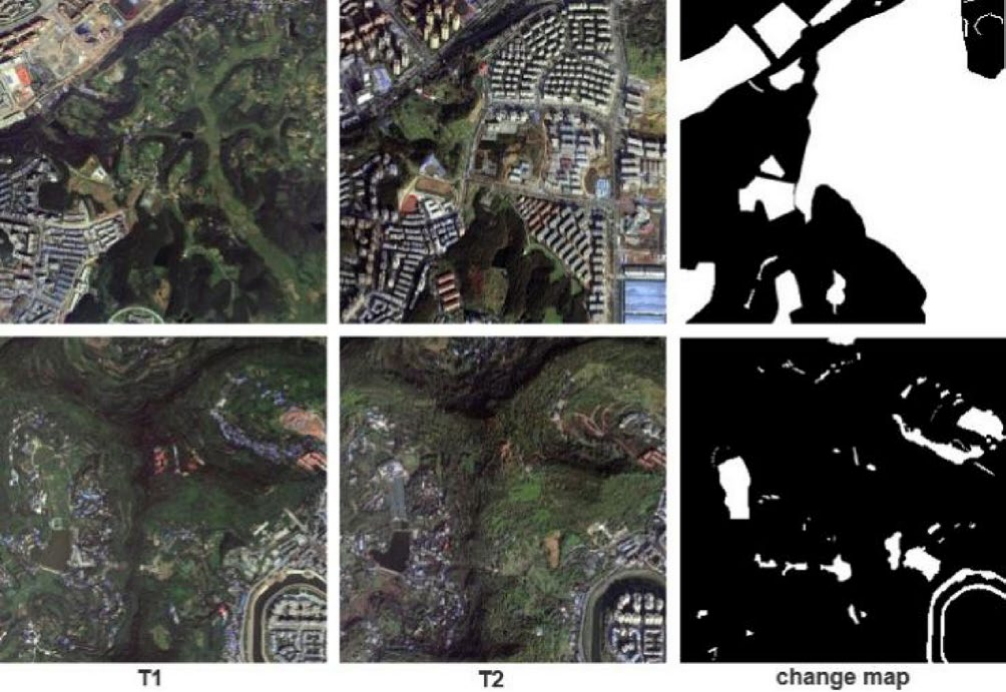
Expansion of the study area toward flat terrain areas.

Areas with lower slopes are suitable for developing urban built-up zones, as such terrain facilitates the construction of roads, buildings, and infrastructure and supports more efficient urban planning, development, and management. In contrast, areas with higher slopes are generally unsuitable for urban expansion due to construction difficulties and potential adverse effects on urban aesthetics and residents’ quality of life. The first characteristic of urban expansion in the study area is the concentration of development in flat areas to meet practical requirements for construction and planning. Over the past decade, in addition to previously under-construction areas transitioning into built-up zones, green spaces with low slope gradients have been largely converted into developed areas, as shown in the first row of Fig. 7. From 2013 to 2022, 89.72% of the newly added construction land in Shunqing District (approximately 25.00 km²) was distributed in the flat areas with a slope of ≤ 8°. The steep slope areas with a slope greater than 15°, as depicted in the second row of Fig. 7, due to the steep terrain, the proportion of newly added construction land in the past decade was less than 1.3%, and there were almost no new roads or permanent buildings. The urban expansion basically stagnated.

#### Expanding along existing roads

**Fig. 8:**
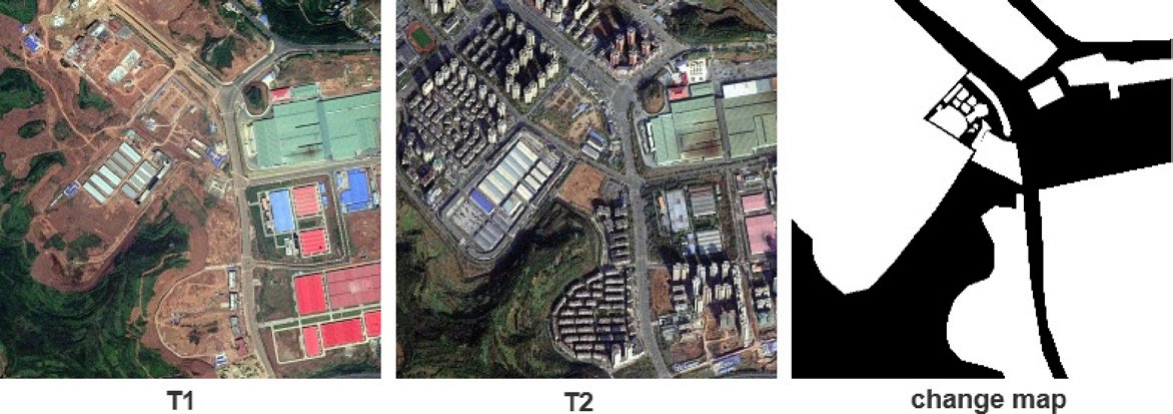
Expansion of the study area along existing roads.

The most straightforward and cost-effective approach to urban expansion is to develop along existing roads. Rational and efficient utilization of road networks enables cities to accommodate additional housing, commercial spaces, and public facilities, thereby meeting residents’ daily needs. Simultaneously, such expansion allows for better use of existing infrastructure, reduces construction costs, and enhances the urban economic environment. Furthermore, development along established roads helps protect historical sites and cultural heritage by minimizing disruptive land use changes caused by uncontrolled sprawl. During periods of rapid urban growth, areas with newly constructed buildings often include accompanying road extensions to serve residential demands. In these cases, construction typically clusters around the new roads. Therefore, expanding along existing transportation corridors not only effectively increases urban scale but also conserves resources and promotes sustainable economic development. In the study area, most regions adjacent to existing roads are densely covered with buildings, as illustrated in Fig. 8. From 2013 to 2022, the transportation network in Shunqing District had a significant influence on the urban spatial layout. 71.35% of the newly built area (19.88 km²) was located within the 500-meter buffer zone of various roads, resulting in a distinct axial expansion pattern of the roads.

#### Expansion along rivers and coasts

**Fig. 9:**
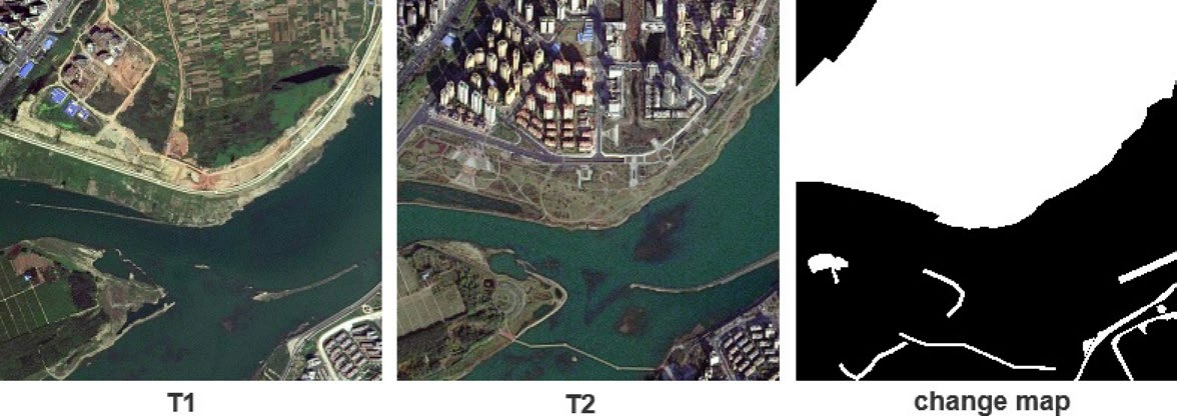
The study area expands along the river and its banks.

Areas along rivers and coasts possess favorable geographical locations and hydrological conditions, making them suitable for residential development. Constructing urban built-up areas in these zones can promote urban economic growth, improve the overall urban environment, and enhance residents’ quality of life. These areas also support comprehensive public facilities such as parks, scenic spots, and cultural amenities, contributing to a more livable urban environment. However, development along rivers and coasts requires scientific planning and effective management to ensure sustainable urban growth while protecting water quality and enhancing the ecological environment. During construction, the characteristics of water conservancy infrastructure, port facilities, and transportation networks must be fully considered, and appropriate measures should be implemented to create functional and aesthetically coherent urban landscapes. In the study area, extensive farmland within the Jialing River Basin has been converted into residential zones. Concurrently, the riparian vegetation landscape has been significantly enhanced. The newly developed residential areas have improved local living conditions, as illustrated in Fig. 9. Shunqing District has formed a typical band-like expansion feature along the main water system of the Jialing River. In the past decade, the area within a 1-kilometer buffer zone along the Jialing River has seen an increase in urban built-up area of 6.93 km², accounting for 24.19% of the total newly added construction land.

#### Expansion along existing urban boundaries

**Fig. 10:**
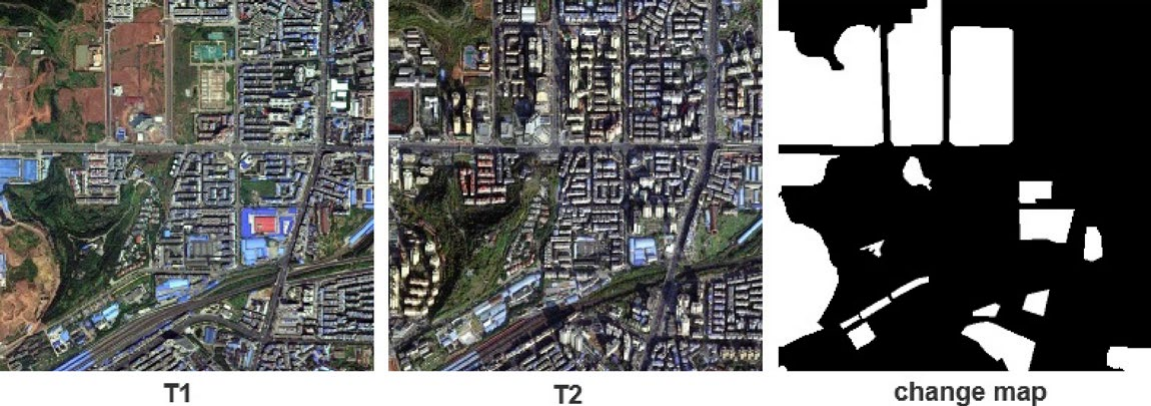
The study area has expanded along the existing urban boundary.

From 2013 to 2022, the urban fringe of Shunqing District added 10.56 km² of new construction land, accounting for 37.90% of the total new construction land. Expansion along existing urban boundaries can be achieved through the following approaches. First, expanding urban land use involves the development and utilization of land through new construction, expansion of existing buildings, and redevelopment of underutilized or vacant areas for alternative purposes. This approach also encompasses the protection of green spaces, improvement of public transportation systems, and effective management of urban sprawl. Second, strengthening urban construction enables peripheral growth by continuously enhancing infrastructure and built environments, thereby extending urban boundaries outward. Third, developing new communities—either as satellite towns linked to the central city via major roads or as independent developments - can contribute to boundary expansion. These communities may be supported by self-contained infrastructure, including housing, commercial and recreational facilities, and transportation networks. In the study area, many residential areas have been newly constructed along existing urban boundaries, leading to outward expansion. Concurrently, the demolition and reconstruction of low-rise industrial zones and outdated rural housing represent a key characteristic of urban expansion in the region, as illustrated in Fig. 10. Over the past decade, the area of inefficient land that has been updated and renovated has reached 3.86 km². This has effectively optimized the internal spatial structure of the city, achieving the urban evolution characteristic of both outward expansion and internal renovation, and continuously expanding the boundaries of urban development.

#### Enhancing the urban living environment

**Fig. 11:**
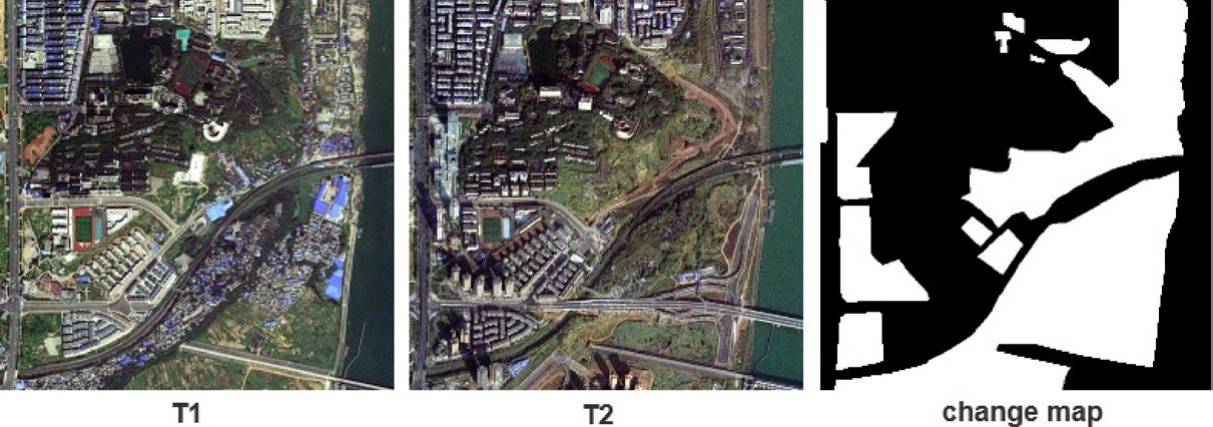
Enhancement of the urban living environment in the study area.

Urban transformations in the study area are also reflected in the continuous improvement of the urban living environment and the enhancement of residents’ quality of life. These improvements include the restoration of riparian vegetation along the riverside road and the construction of parks and other recreational facilities within the city, as illustrated in Fig. 11. The renovation of the riverside road began in 2013, followed by the West River Landscape Corridor project in 2017. Over the past ten years, the urban area has cumulatively added 4.38 km²of ecological green spaces, parks and leisure landscape areas. Vegetation restoration along the river and its banks has contributed to improved soil conditions, enhanced water quality, and a healthier plant ecological environment, while also increasing landscape aesthetic value and residents’ sense of well-being. Additionally, the study area has seen the development of numerous new parks and recreational amenities, including green spaces, pedestrian walkways, and fitness zones, which provide healthy leisure opportunities for urban residents and promote physical activity and social interaction. This type of ecological improvement-oriented construction accounted for 15.29% of the total new land area in the city, these vegetation restoration initiatives have improved air quality and the overall environmental conditions, while the construction of recreational infrastructure has significantly enhanced local living and leisure environments.

### Analysis of urban expansion patterns

#### Balancing resource efficiency and ecological protection

The urban expansion of Shunqing District is constrained by the terrain of shallow hills and river valleys. It is concentrated in the river valley plains and low-lying gentle slopes. It also expands along rivers and along transportation routes in a corridor-like manner, achieving high resource utilization efficiency, but it also brings concentrated pressure on ecological space. It is necessary to coordinate the suitability of the terrain with ecological sensitivity. A buffer zone should be delineated between the development concentration area and the ecological barrier area to achieve a dynamic balance between development intensity and ecological protection, and to ensure the sustainable development of the city.

#### Urban space management and control

The region adopts a core + district group-style and multi-node expansion pattern, with a clear spatial structure. The region adopts a core + district group-based and multi-node expansion pattern, with a clear spatial structure. It should rely on the existing distribution characteristics of the groups, strengthen the boundary control between the core urban area and the peripheral districts, strictly control the continuous expansion of the groups, and divide and control units based on natural elements such as gentle hills and water systems, to stabilize the multi-center spatial structure and enhance the systematicness and precision of spatial governance.

#### Land intensive utilization

The city expands along transportation arteries and river corridors, with strong land utilization orientation and high concentration. Based on this, it is necessary to promote the concentrated and contiguous development of the corridor areas and the river valley and flatland regions along the river. This will revitalize the existing construction land, optimize the layout of the plots, avoid fragmentation and inefficient utilization, and enhance the land utilization efficiency of low hills, gentle slopes and terraces. This will support the orderly expansion of the city through intensive utilization.

#### Ecological wealth

The new construction projects are mainly concentrated in areas along rivers and coasts as well as gentle slopes of low-lying hills. They have a significant impact on the ecological space of the Jialing River and its tributaries, as well as the ecosystem of the hills. It is necessary to strictly implement the control of ecological protection red lines and blue lines, prohibit encroachment on floodplains, high-gradient hillsides and ecological isolation belts, protect ecological corridors and ventilation spaces, and uphold the bottom line of ecological security.

#### Planning and decision-making suggestions

The future urban expansion planning of the research area should conform to the four characteristics of terrain constraints, group structure, riverbank expansion and traffic orientation, and adhere to the multi-center and group-based layout; optimize the functional layout based on the transportation arteries; build an ecological and livable zone relying on the riverfront resources; incorporate the terrain, water systems and ecological red lines into the rigid planning to achieve the goals of adapting to local conditions, developing along the river, and achieving efficient and intensive planning.

## Research limitations and future directions for development

This study validates the effectiveness of deep learning for urban change detection in hilly areas city of western China, yet several technical limitations remain. The change detection model proposed in this study has shown stable performance in monitoring urban expansion in the shallow hilly valley areas of western China. It can effectively identify changes in built-up land and the characteristics of urban spatial expansion. The experimental results indicate that this model is more suitable for monitoring urban expansion in shallow hills and valley areas with similar landform features, similar image resolution, and the same terrain pattern as the study area. It has good applicability in hilly and valley regions with fragmented terrain, scattered settlements, and irregular building distribution. However, due to the sample size and data source type, the model’s promotion capabilities across regions and sensors still have certain limitations.Limited remote sensing data coverage and high annotation costs in the region result in significant dependence on data scale for model training. The complex terrain characterized by mountains and plateaus negatively impacts model generalization, necessitating research into lightweight model deployment on edge devices. Moreover, relying solely on traditional remote sensing data hinders the capture of fine-grained urban dynamics; integrating non-traditional data sources-such as night-time lights and social media-can enhance spatiotemporal resolution. Future studies should advance interdisciplinary integration by incorporating theories from urban economics and sociology to develop a comprehensive technology-society-ecology assessment framework. Establishing a long-term database for sustainable urban development in hilly areas city of western China would enable the identification of change patterns through longitudinal analyses spanning over a decade. Additionally, exploring collaborative detection models within urban agglomerations-such as the Chengdu-Chongqing Twin City Economic Circle-can provide technical support for cross-regional planning coordination.

## Data Availability

The paper uses five public datasets, the first one is OSCD, the second one is CD_Data_GZ, the third one is WHU dataset, the fourth one is Inria dataset, and the fifth one is Massachusetts road dataset. The dataset of Onera Satellite Change Detection (OSCD) is available at https://ieee-dataport.org/open-access/oscd-onera-satellite-change-detection#files.The dataset of Change Detection Data of Guangzhou (CD_Data_GZ) is available at [https://github.com/daifeng2016/Change-Detection-Dataset-for-High-Resolution-Satellite-Imagery](https:/github.com/daifeng2016/Change-Detection-Dataset-for-High-Resolution-Satellite-Imagery). The dataset of WHU (Wuhan University) is available at [http://gpcv.whu.edu.cn/data/index.html](http:/gpcv.whu.edu.cn/data/index.html). The dataset of Inria (Inria Aerial Image Labeling Dataset) is available at [http://pascal.inrialpes.fr/data/human/](http:/pascal.inrialpes.fr/data/human). The dataset of Massachusetts road is available at [http://www.cs.toronto.edu/~vmnih/data/](http:/www.cs.toronto.edu/~vmnih/data). The paper still used two self-constructed datasets, the one is BR_Data_NC, and the other is CD_Data_NC. The dataset of BR_Data_NC (Building and Road Dataset of Nanchong) is available at https://www.kaggle.com/datasets/wanghaiying/buildingroad.The dataset of CD_Data_NC (change detection dataset of Nanchong) is available at [https://www.kaggle.com/datasets/wanghaiying/cd-data-nc](https:/www.kaggle.com/datasets/wanghaiying/cd-data-nc). Source data of this paper is available at [https://www.kaggle.com/code/wanghaiying/urban-change-detection-of-remote-sensing-images](https:/www.kaggle.com/code/wanghaiying/urban-change-detection-of-remote-sensing-images).
